# PRP8-Induced CircMaml2 Facilitates the Healing of the Intestinal Mucosa via Recruiting PTBP1 and Regulating Sec62

**DOI:** 10.3390/cells11213460

**Published:** 2022-11-01

**Authors:** Yuequ Deng, Xiaoqing Xu, Fanze Meng, Jiaqi Lou, Yu Liao, Qi Li, Mengmeng Zhuang, Yong Sun

**Affiliations:** 1 Department of Burn Surgery, The Affiliated Huaihai Hospital of Xuzhou Medical University, Xuzhou 221004, China; 2 Department of Burn Surgery, The 71st Group Army Hospital of PLA, Xuzhou 221004, China

**Keywords:** circMaml2, RNA-binding protein, PTBP1, miR-683, PRP8, burn

## Abstract

Background: Multiple organ dysfunction syndrome (MODS) occurs in the gastrointestinal tract and injured intestinal mucosa is the anatomical basis for various diseases. The expression of circular RNAs (circRNAs) is implicated in many diseases; however, the role of circRNAs in intestinal mucosal injury is yet to be discovered. Our preliminary gene microarray analysis revealed a novel circular RNA, circMaml2, with a significant intestinal mucosal protection effect. Its expression was found to decrease in severely burned intestinal mucosal tissue, whereas its overexpression might facilitate the reconstruction of the injured intestinal mucous membrane. Methods: The function of circMaml2 in cell proliferation and migration was studied in MC38 cells. The repair function of circMaml2 was tested on the intestinal mucosa of mice. RNA-binding protein polypyrimidine tract-binding protein 1(PTBP1) was selected by pull-down assay and mass spectrometry (MS). RNA immunoprecipitation (RIP) was performed to confirm the binding of circMaml2 and PTBP1 and to study PTBP1 and its downstream target, early B-cell factor 1(Ebf1). Bioinformatics software forecast analysis and dual-luciferase reporter assay were performed to ascertain miR-683 and Sec62 as the downstream targets of circMaml2 and miR-683, respectively. Furthermore, PRP8 was discovered to promote the biogenesis of circMaml2. Results: CircMaml2 promotes cell proliferation and migration of MC38 cells and the repair of the intestinal mucosa of mice. This effect is brought about by combining with PTBP1 to improve Ebf1 and interacting with miR-683 to regulate Sec2. Furthermore, PRP8 was discovered to promote the biogenesis of circMaml2. Conclusions: This is the first reported study of the effect of circMaml2 on intestinal mucosal repair.

## 1. Introduction

Burn injury accounts for numerous hospitalizations annually owing to its complexity. Pathogenic bacteria in the host’s intestinal tract can lead to infections following burns. Perturbations of the intestinal barrier following burn injury contribute to the pathology of intestinal infection, intestinal shock, systemic inflammatory syndrome, and even multiple organ dysfunction syndrome [1,2,3,4]. The intestinal barrier function is restored by maintaining an effective circulating blood volume. The rapid identification and control of infection by appropriate antibiotics and optimal nutritional support facilitate treatment of intestinal mucosal injury after burns [5,6,7].

Circular RNAs (circRNAs) were first discovered from plant viruses by Sanger et al. [8]; however, most studies focused on studying linear RNA and the splicing technique has just been developed. Therefore, circRNAs have not been studied extensively. Moreover, because of the lack of a 3′-terminal polyadenylate tail, most circRNAs cannot be detected by ordinary RNA high-throughput sequencing (RNA-seq). RNA-seq, though, can identify “back-to-back splicing” sites and has discovered thousands of circRNAs from transcripts of humans, mice, and other species [9,10]. Given the special structure of circRNAs, novel detection and quantitation methods, such as specific circRNA bioinformatics algorithms, have been discovered. Moreover, the tissue and cell specificity of circRNAs have also been studied [11,12,13,14]. For example, circTLK1 plays a critical role in renal cell carcinoma [15] and circNRIP1 acts as a tumor promoter in gastric cancer [16,17]. In our previous study, circMaml2 expression was attenuated in the intestinal tissue of mice subjected to burn damage. CircMaml2 is a 1686 nt circular transcript whose gene is located on chromosome 9. Its role in reconstructing burn-impaired intestinal mucosa of mice has not been studied.

CircRNAs regulate transcription, splicing, and chromatin formation in the nucleus. Upon entering the cytoplasm, circRNAs can bind to microRNA (miRNA) and, as an endogenous competitive RNA (ceRNA), inhibit the binding between miRNA and its downstream target mRNA. They do so by competing for shared microRNA response elements and binding to the 3′-untranslated region (3′UTR), inhibiting the translation of target genes regulated by the miRNAs [17,18,19]. Additionally, circRNAs can bind to RNA-binding proteins (RBPs), a class of ubiquitously expressed proteins that regulate basic cellular activities by acting on the nucleoprotein complex (RNP) [20]. Some RBPs regulate the reverse splicing and formation of circRNAs by binding to circRNAs through their double-stranded RNA-binding domains [21]. RBPs without these domains can also regulate the production of circRNAs by directly binding to specific RNA motifs.

Furthermore, circRNAs bound to RBPs are involved in cell cycle progression and the expression of circRNA mother genes that regulate the biogenesis of circRNAs [22,23]. CircRNAs are also involved in protein translation [24,25]. The earliest link between circRNAs and disease pathology was an accidental discovery of transcripts produced by non-classical splicing from colon cancer genes. Subsequently, circRNAs have been shown to contribute to various diseases.

In this study, we found a significant decrease in the expression of circMaml2 both in vitro in mouse intestinal epithelial (MC38) cells and in vivo in the burn-damaged intestinal tissue of mice, implying cell proliferation and migration could be promoted by circMaml2 overexpression. CircMaml2 interacts with the RBP PTBP1 and regulates the expression of Ebf1. Additionally, circMaml2 sponges miR-683 and indirectly regulates Sec62 expression. Thus, circMaml2 promotes cell proliferation and migration, facilitating the reconstruction of the intestinal mucosa via these two pathways. Furthermore, the biogenesis of circMaml2 was found to occur through PRP8. In short, circMaml2 could be a crucial molecular marker in the intestinal mucosa after severe burns for possible diagnosis and gene therapy.

## 2. Materials and Methods

### 2.1. Cell Lines

The MC38 line was obtained from the GuangZhou Jennio Biotech Co., Ltd. (Guangzhou, China). It was cultured in RPMI 1640 medium (Gibco, Shanghai, China, 2122752) with 10% fetal bovine serum (FBS) (Clark Bioscience, Shanghai, China), 100 U/mL penicillin, and 100 µg/mL streptomycin at 37 °C and 5% CO_2_. The hypoxia condition in this experiment was 1% O_2_, 5% CO_2_, and 94% N_2_.

### 2.2. RNase R Treatment

The cells were incubated with RNase R treatment solution with 1 U/µg of RNase R (Epicenter, WI, USA) at 37 °C for 15 min, and circMaml2 and Maml2 mRNA levels were detected by qRT-PCR.

### 2.3. RNA Extraction, gDNA Extraction, and qRT-PCR Analysis

TRIzol (Thermo, Shanghai, China) was used to extract the total RNA from tissues or cells, followed by reverse transcription using the FastKing RT kit (with gDNase) and miRcute miRNA first-strand cDNA synthesis kit (Tiangen Biotechnology, Beijing, China). The reverse transcription samples were then added to 2X Universal SYBR Green Fast qPCR Mix (ABclonal Technology Co., Ltd., Wuhan, China) and processed with the Light Cycler 480 system (Roche, Basel, Switzerland) for real-time fluorescence quantitative PCR. Finally, the results were calculated by the 2−ΔΔCT method. All primers used in this study, including internal references, are listed in Table 1.

### 2.4. Nucleic Acid Electrophoresis

The agarose solution (1.5%) was melted and then poured into the mold and cooled. The complementary DNA (cDNA) and gDNA PCR products were separated and identified by agarose gel electrophoresis.

### 2.5. Experimental Animals and Establishment of the Burn Model

We used healthy C57BL/6J adult mice to establish the burn model. The Animal Ethics Committee approved the experimental protocol of Xuzhou Medical University. The mice were acclimated for 1 week and randomized into five groups: control group (*n* = 10), control + circMaml2 NC group (*n* = 10), control + circMaml2 group (*n* = 10), burn group (*n* = 10), and burn + circMaml2 group (*n* = 10) before initiation of any experimental procedures. Mice in the control + circMaml2 NC group, control + circMaml2 group and burn + circMaml2 group were intraperitoneally injected with CircMaml2 adenovirus or CircMaml2 NC adenovirus (Shanghai Hanheng Biotechnology, Shanghai, China) (10 µL/g) before the beginning of the actual experiment. First, the mice were anesthetized by intraperitoneal injection of pentobarbital sodium (40 mg/kg). The fur on the back of the mice was removed to expose at least 30% of the whole-body area to ensure enough burn severity. The bare skin was exposed to 100 °C water for 8 s until 30% third-degree scalding was achieved. The burned mice were injected intraperitoneally with lactic acid green sodium (50 mL/kg). The mice were sacrificed after 72 h, followed by dissection of the intestinal tissue.

### 2.6. H&E Staining

A part of the intestinal tissue was fixed in 10% formalin solution and 3 µm thick sections were taken and observed under light microscopy. The sections were assessed according to the standard shown in Table 2.

### 2.7. Adenovirus Infection

The recombinant adenovirus and scrambled control (NC) were obtained from Shanghai Hanheng Company. The GeneBank accession number of circMaml2 is NR_033241.1. Adenovirus (MOI = 300) was transfected into cells incubated in half volume of RPMI-1640 with 10% FBS for 4 h, and the other half of RPMI-1640 was added to the first half. After another 4 h, the medium was replaced with a new conventional medium and the infected cells were cultured for about 36–48 h until the expression level of circMaml2 was detected.

### 2.8. Colony Formation Assays

After diluting the cell suspension, each group of cells with 100 cells/mL was inoculated in a culture medium in a petri dish and placed in an incubator for 2–3 weeks until colony formation (>50 cells per colony) was observed. The samples were fixed with 4% paraformaldehyde and stained with 0.5% crystal violet.

### 2.9. Cell Counting Kit-8 (CCK-8) Assays

MC38 cells were grouped and incubated for 0, 24, 48, 72, and 96 h in five 96-well plates. At each time interval, 10 µL CCK8 solution (Meilunbio, Dalian, China) was added to each well and incubated for 2 h. The absorbance of the cells was measured at 450 nm using a multifunctional microporous plate detector (Synergy, Boston, MA, USA). Wells without cells were used as the blank control group. 

### 2.10. Edu Assays

The prepared cells were processed using the Cell-Light EdU Apollo567 In Vitro kit (Ribobio, Guangzhou, China) according to the protocol provided by the manufacturer to assess the cell proliferation based on the ratio of EdU-stained cells to Hoechst-stained cells.

### 2.11. Wound Healing Assays

Cells at 100% confluence were used in the wound healing assay. An artificial wound was created using a sterile 10 µL pipette tip. The cells were incubated in a serum-free medium until 100% confluence was obtained. The wound area was observed and photographed during the treatment at 0, 24, and 48 h. Before the creation of an artificial wound, mitomycin C was added to the prepared cells with the final concentration of 5 µg/mL and incubated for 2 h.

### 2.12. Transwell Assays

An appropriate volume of cell suspension was added into the upper layer of the chamber, while 600 µL of 1640 medium with 20% FBS was added into the lower layer of the chamber. Later, the 24-well plate was incubated for 48 h, fixed with 4% paraformaldehyde, and stained with 0.5% crystal violet. Cells in the upper chamber were gently wiped with a cotton swab.

### 2.13. RNA Isolation of Nuclear and Cytoplasmic Fractions

The Minute^TM^ Cytoplasmic and Nuclear Isolation kit (Invent Biotechnologies, Beijing, China) was used to separate the nuclear and cytoplasmic material of the cells according to the manufacturer’s protocol. After RNA extraction, circMaml2 expression in the nucleus and cytoplasm was detected by qRT-PCR; U6 was used as the internal control for the nuclear sample, whereas Gapdh was used as the control for the cytoplasmic sample.

### 2.14. Pull-Down Assay and MS Analysis

We carried out an RNA pull-down experiment to determine the RBP binding to circMaml2 using MS2 binding protein (MS2bp). The RNA-containing MS2-binding sequence (MS2bs) of MS2 was specifically bound to circMaml2. A construct containing the bound circMaml2 transcript and MS2bs element was generated and co-transfected into MC38 cells along with the construct containing MS2bp Flag. The cell samples were processed using lysate, protease inhibitor, and RNase inhibitor. Magnetic beads were added to bind the antibody. Next, binding buffer and cell lysate were added to capture the antigen. Finally, protein products from the pull-down samples were extracted and analyzed by MS.

### 2.15. RNA Immunoprecipitation (RIP)

RIP experiments were performed using the RNA Immunoprecipitation kit (BersinBio, bes5101, Guangzhou, China). After adding the polysome analysis buffer to the cells, the cells were freeze-thawed and DNase salt stock and DNase were used to remove DNA. The cell lysis samples were divided into IP, IgG, and input. The input samples were stored at −80 °C for standby, whereas the IP and IgG samples were added to the experimental antibodies for overnight incubation. The magnetic beads were balanced using polysome lysis buffer during incubation. The balanced magnetic beads were added to the incubated samples, which were further incubated for 1 h. After repeated washing, the RNA was eluted and the RNA extract was added. The subsequent steps were the same as those for total RNA extraction, reverse transcription, and real-time fluorescence quantitative PCR.

### 2.16. Western Blot Analysis

RIPA lysate and protease inhibitor (Beyotime Biotechnology, Shanghai, China) was added in the ratio of 100:1 to the cells on ice. The obtained protein samples were analyzed and balanced according to the BCA protein determination kit instructions. The proteins, after boiling, were separated by sodium dodecyl sulfate-polyacrylamide gel electrophoresis (Yeasen Biotechnology, Shanghai, China) and electroblotted on a polyvinylidene fluoride membrane (Millipore, Boston, MA, USA). The strip was sealed using milk for at least 2 h and incubated overnight with primary antibodies, followed by the secondary antibody for 2 h. The strip was scanned using the dual infrared laser imaging system (Odyssey, Shanghai, China). Densitometric analysis was performed using ImageJ.

### 2.17. Lentivirus Transfection

The Shanghai Hanheng Company constructed the lentivirus and scrambled control (NC) vectors. Cells were transfected by lentivirus (MOI = 10) exposure in half volume of RPMI-1640 supplemented with 5 µg/mL polybrene for 4 h and another 4 h with the other half of RPMI-1640 with 10% FBS. After 24 h of transfecting the medium with the virus, the medium was replaced with a conventional medium. The infected cells were cultured for about 48–72 h until the green fluorescence could be observed using a fluorescence microscope. The medium was then replaced with a medium containing 10% FBS and 6 µg/mL puromycin and incubated until the transfection of cells was stable.

### 2.18. Assessment of Endogenous RNA Degradation

Cells in the logarithmic growth stage were cultured with actinomycete D (MedChemExpress, Princeton, NJ, USA; 0.5 mg/L). RNA was extracted from the control and experimental groups at 0, 30 min, 1 h, 2 h, 4 h, 8 h, 12 h, and 24 h. The expression of downstream mRNA was detected by real-time fluorescence quantitative PCR and the amount of mRNA remaining was compared to that at 0 h.

### 2.19. Transfection of siRNA and miRNA Mimic

The miRNA mimic or siRNA was transfected into the prepared cells using X-tremeGENE siRNA Transfection Reagent (Roche, Basel, Switzerland). Total RNA was extracted from the cells after 48 h of incubation, while the proteins were extracted after 72 h. Finally, the expression of miR-683 or Ebf1 was detected using qRT-PCR.

### 2.20. Dual-Luciferase Reporter Assay

The bioinformatic websites miRWalk and TargetScan were used to predict the binding between circMaml2 and miR-683 and between miR-683 and Sec62. The results were verified by performing a dual-luciferase reporter gene experiment according to the predicted binding sites obtained from the bioinformatic websites miRanda and TargetScan. Wild-type and mutant circMaml2 transcripts (NR_033241.1) and Sec62 3′-UTR were introduced into the psiCHECK-2 luciferase reporter vector (Promega, Madison, WI, USA) and co-transfected with the miR-683 mimic or NC into MC38 cells. Finally, the relative luciferase activity was assessed by the dual-luciferase reporter gene detection kit (Beyotime, Shanghai, China).

### 2.21. Statistical Analysis

SPSS 22.0 (IBM, SPSS, Chicago, IL, USA) was used for data analysis and GraphPad Prism 9.0 (GraphPad Software, San Diego, CA, USA) was used for preparing the graphs. All values are expressed as the mean ± standard deviation. The Student’s *t*-test was used to compare two groups and a one-way or two-way analysis of variance was used to compare multiple groups. *p* < 0.05 was considered to indicate statistical significance. All experiments were conducted at least in triplicates.

## 3. Results

### 3.1. CircMaml2 Upregulation Promotes the Migration and Proliferation of MC38 Cells

**Figure 1:**
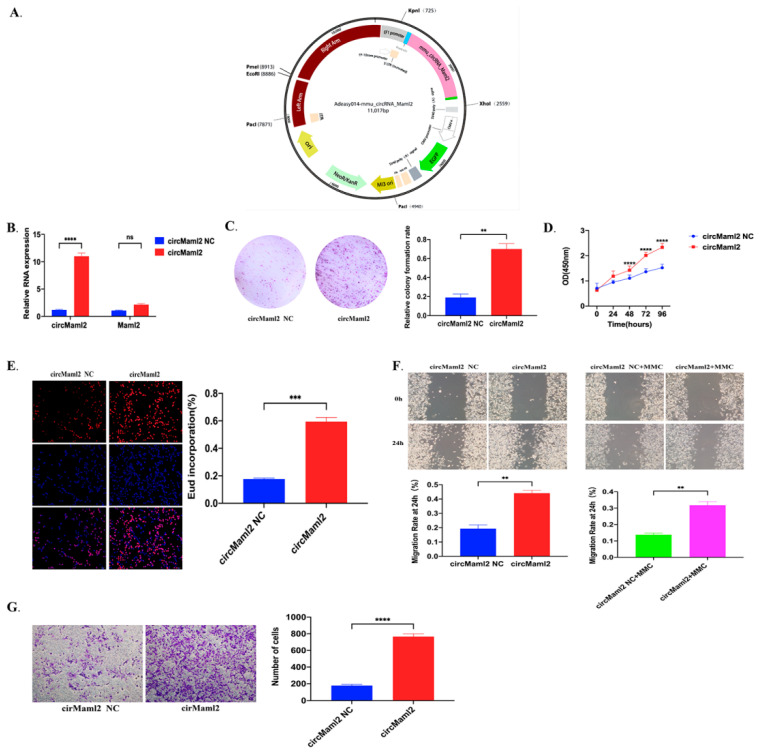
CircMaml2 Upregulation Promotes the Migration and Proliferation of MC38 Cells (**A**) A schematic of the structure and sequence of the adenovirus construct of circMaml2. (**B**) qRT-PCR assays were conducted to confirm increased expression of circMaml2 after transfection with adenovirus targeting circMaml2 (**** *p* < 0.0001, ns *p* = 0.0789, two-way ANOVA). (**C**) A colony formation assay was performed to examine the role of CircMaml2 in cell proliferation (** *p* = 0.0018, *t* test). (**D**) Growth curves of MC38 cells transfected with Ad-circMaml2 detected by the CCK-8 assay (**** *p* < 0.0001, two-way ANOVA). (**E**) The number of EdU-positive cells detected by the EdU assay in MC38 cells transfected with Ad-circMaml2 (*** *p* = 0.0002, *t* test). (**F**,**G**) Migration of MC38 cells transfected with Ad-circMaml2 detected by wound healing and transwell experiments using (or not using) the MMC preconditioning (** *p* = 0.0018, ** *p* = 0.0016, **** *p* < 0.0001, *t* test). All data are presented as mean + SEM. ns, not significant. *n* = 3.

To further explore the biological significance of circMalm2 in burn progression precisely, we constructed the pAdEasy-EF1-MCS-CMV-GFP adenovirus overexpression vector with circMaml2. CircMaml2 could be expressed efficiently by the CMV promoter of human cytomegalovirus (Figure 1A). In this vector, the upstream and downstream loop forming elements, Front circ-Signal and Back circ-Signal, can promote the loop of expressed circRNA effectively, whereas the linearized vector could cause direct cloning of circRNA with little unrelated sequences participating in a ring formation. We transfected the adenovirus overexpression vector with circMaml2 into the MC38 cell line and the vector was detected to be expressing stably in the cell line. The expression of linear Maml2 mRNA was not altered by the overexpression of circMaml2 (Figure 1B). Furthermore, circMaml2 remarkably increased the number of colonies of MC38 cells (Figure 1C). The results of the CCK-8 assay showed that circMaml2-overexpression could significantly promote the proliferation of MC38 cells (Figure 1D). The number of EdU-positive cells was increased by circMaml2 upregulation (Figure 1E). Moreover, wound healing and Transwell assays verified that circMaml2 promotes the migration of MC38 cells. In wound healing assays, MMC (mitomycin C) was utilized to precondition cells to avoid the effect of cell proliferation. As a result, it was discovered that, in the group of circMaml2 NC, cells had migrated to some extent comparing 0 h with 24 h, but in the group of circMaml2 NC + MMC, the cell migration was inhibited apparently. However, it was manifested that circMaml2 could obviously promote cell migration whether using MMC or not (Figure 1F,G).

### 3.2. CircMaml2 Directly Interacts with the RBP PTBP1

**Figure 2:**
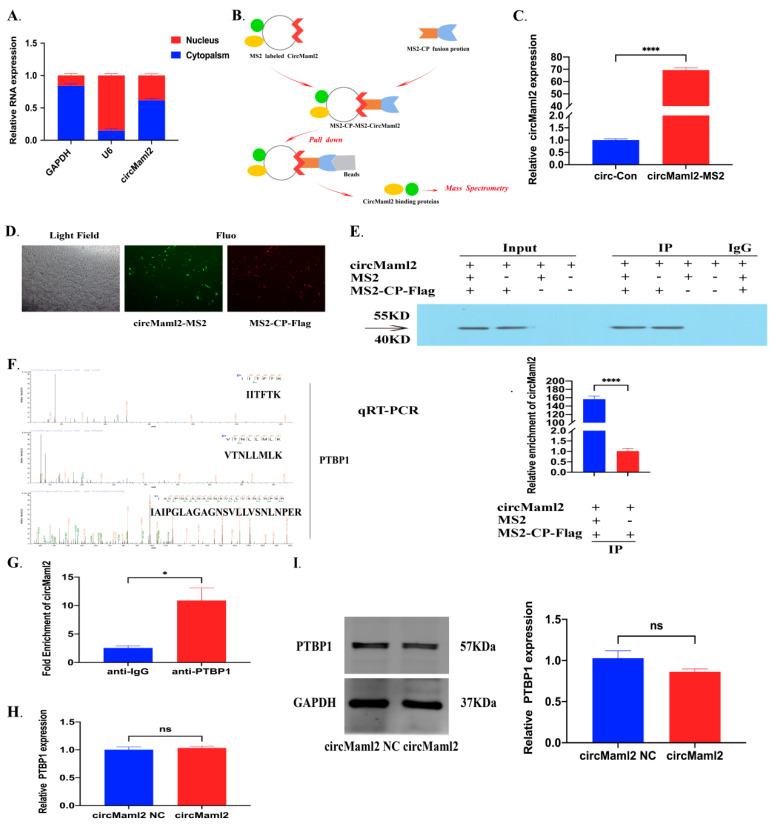
CircMaml2 Directly Interacts with the RBP PTBP1. (**A**) Subcellular location of circMaml2 in MC38 cells. (**B**) In vivo circRNA pull-down using the MS2-tagging system and subsequent label-free mass spectrometric analysis (MS). (**C**) Confirmation of the overexpression of circMaml2 using qRT-PCR analysis (**** *p* < 0.0001, *t* test). (**D**) Co-transfection of circMaml2-MS2 and MS2-CP-Flag plasmids to induce the expression of MS2 RNA hairpins with overexpressed circMaml2 and a fusion protein MS2-CP-Flag, which could recognize MS2 RNA hairpins. The green fluorescence-labeled circMaml2 (up) and the red fluorescence-labeled MS2-CP-Flag (bottom). (**E**) Western Blot test the MS2-CP-Flag pulled down by anti-Flag (up). The enrichment of circMaml2 in the complex with MS2-CP-Flag was detected by qRT-PCR (bottom) (**** *p* < 0.0001, *t* test). (**F**) A label-free MS was used to test the protein complex with MS2-CP-Flag. The peptides were matched to PTBP1. (**G**) The immunoprecipitation assay (RIP) measures the amount of circMaml2 pulled down by PTBP1 and IgG antibodies in MC38 cells (* *p* = 0.0196, *t* test). (**H**,**I**) Both qRT-PCR and Western blot detected PTBP1 expression in circMaml2-overexpressing cells. All data are presented as mean + SEM. ns, not significant. *n* = 3.

**Figure 3:**
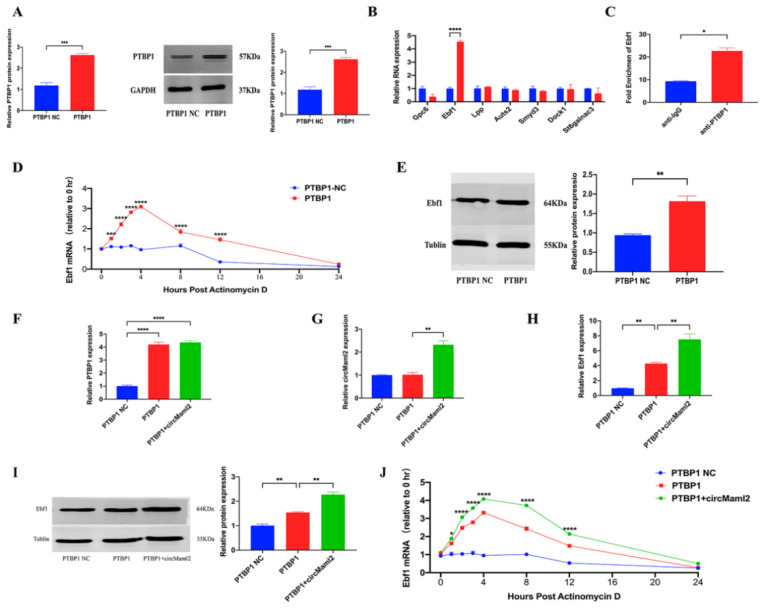
CircMaml2 Enhances PTBP1 to Upregulate Ebf1 Expression. (**A**) Verification of the overexpression of PTBP1 by lentivirus in MC38 cells though qRT-PCR (left) and Western blot (right) (*** *p* = 0.0008, *t* test). (**B**) The expression level of downstream target mRNAs after overexpression of PTBP1 (**** *p* < 0.0001, two-way ANOVA). (**C**) Using anti-IgG and anti-PTBP1 antibodies. The percentage of RIP-enriched PTBP1 and Ebf1 mRNA relative to the input value was calculated by qRT-PCR (* *p* = 0.0104, *t* test). (**D**) qRT-PCR was used to measure the expression levels of Ebf1 after actinomycin D treatment in the context of PTBP1 overexpression (*** *p* = 0.0004, **** *p* < 0.0001, two-way ANOVA). (**E**) Protein expression of Ebf1 upon PTBP1 overexpression in MC38 cells (** *p* = 0.0031, *t* test). (**F**,**G**) Verification of Ad-circMaml2 in PTBP1 overexpression MC38 cells (**** *p* < 0.0001, ordinary one-way ANOVA; ** *p* = 0.0029, *t* test). (**H**,**I**) The expression of Ebf1 was detected by qRT-PCR and Western blot after transfecting Ad-circMaml2 in PTBP1 overexpression MC38 cells (** *p* = 0.0044, ** *p* = 0.0051, ** *p* = 0.0056, ** *p* = 0.0012, ordinary one-way ANOVA). (**J**) qRT-PCR was used to measure the expression levels of Ebf1 after actinomycin D treatment in the context of PTBP1 and circMaml2 overexpression meantime (* *p* = 0.0411, **** *p* < 0.0001, two-way ANOVA). All data are presented as mean + SEM. ns, not significant. *n* = 3.

The nuclear-cytoplasmic separation test confirmed that circMaml2 was located in the cytoplasm (Figure 2A). For the RNA pull-down assay to identify the possible RBPs binding to circMaml2, we first constructed a vector of circMaml2 labeled with MS2 (circMaml2-MS2), which was co-transfected into MC38 cells with MS2-CP fusion protein, and MS2-CP-MS2-circMaml2 was bonded using beads to pull-down RBPs interacting with circMaml2 (Figure 2B). The circMaml2-MS2 complex was then detected by qRT-PCR (Figure 2C). Next, two plasmids were constructed expressing circMaml2-MS2 and MS2-CP-Flag, respectively, and co-transfected into MC38 cells (Figure 2D). The MS2-CP-MS2-circMaml2 complexes were pulled down using Flag antibodies (Figure 2E, top panel). The qRT-PCR analysis also provided the same result (Figure 3E, bottom panel), which demonstrated the specificity of our pull-down isolation. Lastly, three peptides bound to circMaml2 were identified and matched with PTBP1 (Figure 2F). The RIP assay conducted in MC38 cells showed that circMaml2 pull-down increased after anti-PTBP1 immunoprecipitation compared with IgG (Figure 2G). These results validated the interaction between circMaml2 with PTBP1. Furthermore, we observed that overexpression of circMaml2 did not significantly affect the expression of PTBP1, demonstrating that circMaml2 may physically bind to PTBP1 without affecting PTBP1 expression (Figure 2H,I).

### 3.3. CircMaml2 Enhances PTBP1 to Upregulate Ebf1 Expression

To explore further the mechanism of PTBP1 downstream, we constructed and verified a specific PTBP1 lentivirus overexpression vector in MC38 cells (Figure 3A). The downstream mRNA targets of PTBP1 were predicted using the website http://starbase.sysu.edu.cn (accessed on 29 August 2022), including Gpc6, St6galnac3, Ebf1, LPP, Auts2, Smyd3, and Dock1. qRT-PCR showed that the expression of Ebf1 increased steadily with PTBP1 overexpression (Figure 3B). RIP assay demonstrated that the pull-down of Ebf1 was enriched after anti-PTBP1 immunoprecipitation compared with IgG (Figure 3C). The actinomycin D experiment to test the mRNA stability showed that under the same conditions, the levels of Ebf1 mRNA on PTBP1 overexpression were higher at each time point on the addition of actinomycin D (Figure 3D). Western blot analysis also exhibited that PTBP1 promotes Ebf1 expression (Figure 3E). Furthermore, to demonstrate the interaction between circMaml2 and PTBP1, we transfected Ad-circMaml2 into the PTBP1 overexpression MC38 cells and verified the expression by qRT-PCR (Figure 3F,G). qRT-PCR and Western blot analysis showed that the expression of Ebf1 decreased in the following order: PTBP1 + circMaml2 group > PTBP1 group > control group. Thus, it can be speculated that circMaml2 improves the function of PTBP1 (Figure 3H,I). The same result was seen in the actinomycin D experiment (Figure 3J).

### 3.4. CircMaml2 Promotes the Migration and Proliferation of MC38 Cells by Acting on PTBP1-Ebf1

**Figure 4:**
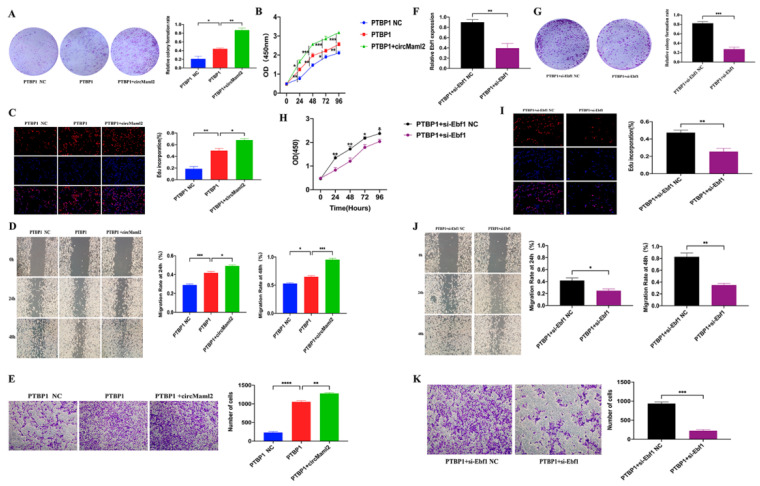
CircMaml2 Promotes the Migration and Proliferation of MC38 Cells by Acting on PTBP1-Ebf1. (**A**) Proliferation of cells was examined using colony formation assays (* *p* = 0.0391, ** *p* = 0.0019, ordinary one-way ANOVA). (**B**) The CCK-8 assay detects growth curves of PTBP1 overexpression MC38 cells transfected with Ad-circMaml2 (24 h ** *p* = 0.0038, * *p* = 0.0109; 48 h ** *p* = 0.0021, *** *p* = 0.0004; 72 h * *p* = 0.0442, *** *p* = 0.0002, 96 h ** *p* = 0.005, *** *p* = 0.0003, ordinary one-way ANOVA). (**C**) EdU assay detected a number of EdU-positive cells (** *p* = 0.0024, * *p* = 0.0349, ordinary one-way ANOVA). (**D**,**E**) The migration ability was detected using wound healing experiment and transwell experiment (24 h *** *p* = 0.001, * *p* = 0.0168; 48 h * *p* = 0.0161, *** *p* = 0.0001; **** *p* < 0.0001, ** *p* = 0.0026, ordinary one-way ANOVA). (**F**) Expression of Ebf1 was determined using qRT-PCR ** *p* = 0.0084, *t* test. (**G**) Colony formation assays were performed to examine cells’ proliferation (*** *p* = 0.0006, *t* test). (**H**) Growth curves were detected by the CCK-8 assay (24 h ** *p* = 0.0032, 48 h ** *p* = 0.0029, 72 h * *p* = 0.0201, 96 h * *p* = 0.0458, two-way ANOVA). (**I**) Number of EdU-positive cells was detected by the EdU assay (** *p* = 0.0093, *t* test). (**J**,**K**) Using wound healing experiments and transwell experiments, migration ability was detected (24 h * *p* = 0.0336, 48 h ** *p* = 0.0024, *** *p* = 0.0002, *t* test). All data are presented as mean + SEM. ns, not significant. *n* = 3.

The colony formation assay and the CCK-8 assay verified that circMaml2 regulates the proliferation of MC38 cells via PTBP1. Cell proliferation in the PTBP1 + circMaml2 group was significantly increased, which meant that circMaml2 could significantly promote the function of PTBP1 (Figure 4A,B). The upregulation of circMaml2 and PTBP1 significantly increased the number of EdU-positive cells compared with PTBP1 overexpression alone (Figure 4C). Simultaneously, wound healing and Transwell analyses showed that the overexpression of circMaml2 could significantly promote the migration of PTBP1 overexpressed cells (Figure 4D,E). To verify the link between PTBP1 and Ebf1, si-Ebf1 was transfected into MC38 cells with already overexpressed PTBP1 and qRT-PCR analysis was performed (Figure 4F). The colony formation assay showed that, after Ebf1 knockdown, the positive effect of PTBP1 on cell proliferation was lost (Figure 4G). The CCK-8 assay also verified this result (Figure 4H). Moreover, the EdU assay revealed that the knockdown of Ebf1 significantly decreased the number of EdU-positive cells (Figure 4I). The wound healing experiment suggested that Ebf1 knockdown along with PTBP1 overexpression could reduce wound healing compared with PTBP1 overexpression alone (Figure 4J). The transwell assay also showed the same result (Figure 4K).

### 3.5. CircMaml2 Sponges miR-683

**Figure 5:**
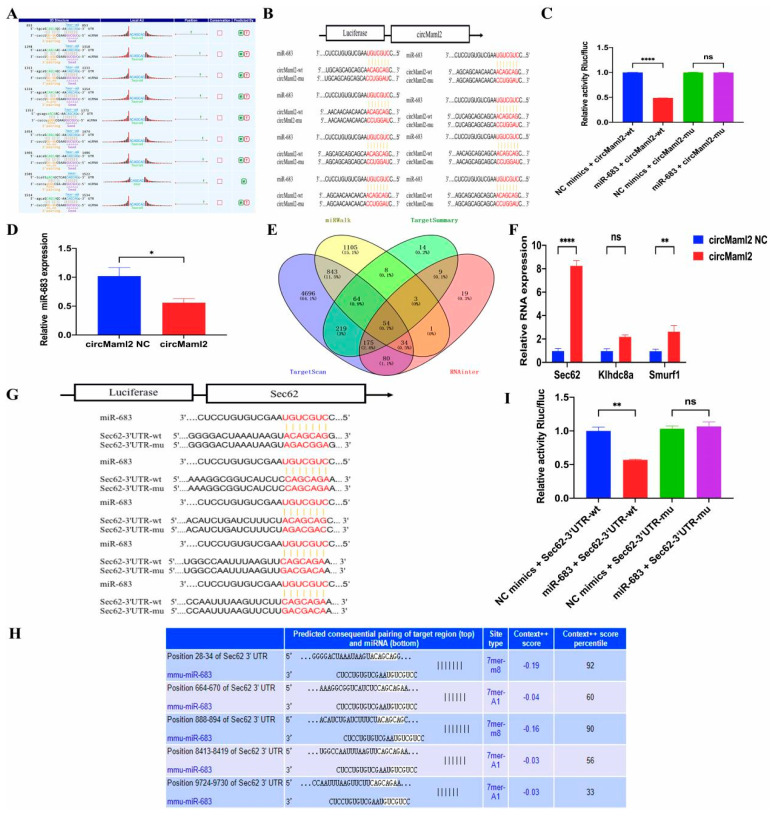
CircMaml2 Sponges miR-683. (**A**) Bioinformatics software predicted the binding sites for circMaml2 and miR-683. (**B**) Schematic illustration of circMmal2-WT and circMmal2-Mut luciferase reporter vectors. (**C**) Relative luciferase activities were investigated in MC38.wt cells after transfection with circMmal2-WT or circMmal2-Mut and miR-683 mimics or miR-NC (**** *p* < 0.0001, ordinary one-way ANOVA). (**D**) Relative miR-683 expression after circMaml2 overexpression (* *p* = 0.0494, *t* test). (**E**) Fifty four possible genes of functional targets of miR-683 were indicated by a Venn diagram. (**F**) MiR-683 dramatically inhibited Sec62 was detected by qRT-PCR. (**G**) TargetScanMouse was used to predict the sequence between miR-683 and Sec62. (**H**) The mutant binding sites of miR-683 at the 3′ UTR of Sec62 mRNA. (**I**) Relative luciferase activities of Sec62−3′ UTR-wt or Sec62−3′UTR-mut were analyzed (*n* = 3) (** *p* = 0.0011, ordinary one-way ANOVA). All data are presented as mean + SEM. ns, not significant. *n* = 3.

CircRNAs can also act as ceRNAs to sponge miRNAs. To investigate this property of circMaml2, we performed bioinformatic analysis to predict the potential miRNAs that may bind to circMaml2 and determine their corresponding bind sites (Figure 5A). We cloned the hypothetical miR-683 target-binding sequence into a luciferase construct to verify whether circMaml2 directly regulates miR-683 (Figure 5B). MiR-683 mimics significantly reduced the luciferase activity of the circMaml2 wild-type reporter gene, but not of the mutated circMaml2, verifying that circMaml2 combines with miR-683 (Figure 5C). The QRT-PCR results showed that circMaml2 negatively regulates the expression of miR-683 (Figure 5D) because circRNAs act as a ceRNA and compete with mRNA for miRNA binding [26,27]. Four bioinformatics tools (miRWalk, TargetSummary, TargetScan, and RNAInter) were used to predict 54 genes that may be biological targets of miR-683 (Figure 5E). Out of these, three with relevant functions were selected, and Sec62 was then investigated in this study by qRT-PCR analysis (Figure 5F). TargetScan was used to identify the potential binding sites between miR-683 and Sec62 (Figure 5G). Similarly, we constructed the Sec62-3′ UTR-wild and Sec62-3′ UTR-mut reporter gene vectors (Figure 5H). The miR-683 mimic reduced the relative luciferase activity of Sec62-3′ UTR-wild-type, but not Sec62-3′ UTR-mut (Figure 5I).

### 3.6. CircMaml2 Rescues the Inhibition of miR-683

**Figure 6:**
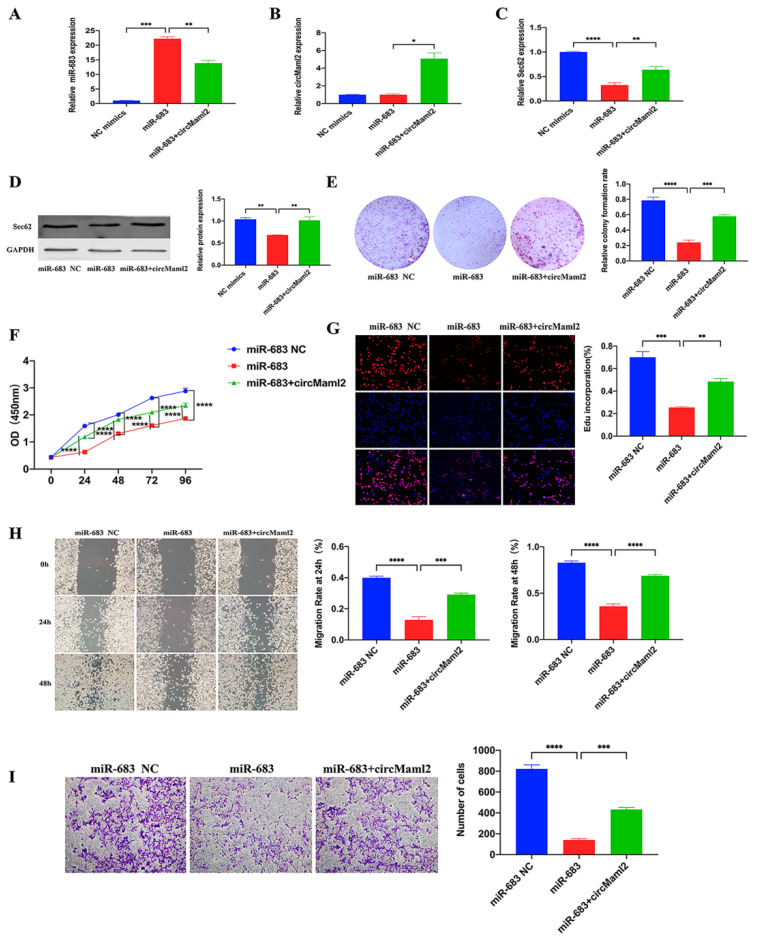
CircMaml2 Rescues the Inhibition of miR-683. (**A**,**B**) Expression of miR-683 and circMaml2 after the up-regulation of miR-683 and circMaml2 was detected by qRT-PCR (*** *p* = 0.0004, ** *p* = 0.0058, * *p* = 0.0113, ordinary one-way ANOVA). (**C**,**D**) mRNA and protein expression of Ebf1 was reversed successfully by co-transfection of circMaml2 and miR-683 (**** *p* < 0.0001, ** *p* < 0.0062, ** *p* = 0.0059, ** *p* = 0.0081, ordinary one-way ANOVA). (**E**–**I**) The cell proliferation and migration ability was rescued by circMaml2 (**** *p* < 0.0001, *** *p* < 0.0006, ordinary one-way ANOVA; **** *p* < 0.0001, two-way ANOVA; *** *p* = 0.0002, ** *p* = 0.0064, **** *p* < 0.0001, *** *p* = 0.0005, **** *p* < 0.0001, *** *p* = 0.0005, ordinary one-way ANOVA). All data are presented as mean + SEM. ns, not significant. *n* = 3.

To investigate if circMaml2 influences the function of miR-683 by acting as a ceRNA, we co-transfected the two together in MC38 cells and detected their expression by qRT-PCR (Figure 6A,B). MiR-683 significantly downregulated the expression of Sec62 at the mRNA and protein levels, but the addition of circMaml2 reversed the inhibition (Figure 6C,D). Colony formation assays, CCK8 analysis, and the EdU assays revealed that the miR-683 mimics significantly inhibited cell proliferation, whereas circMaml2 reversed the negative results (Figure 6E–G). The wound healing and transwell assays showed that miR-683 mimics significantly impeded cell migration, whereas circMaml2 successfully reversed the reduction in cell migration caused by miR-683 (Figure 6H,I).

### 3.7. PRP8 Induces the Biogenesis of CircMaml2

**Figure 7:**
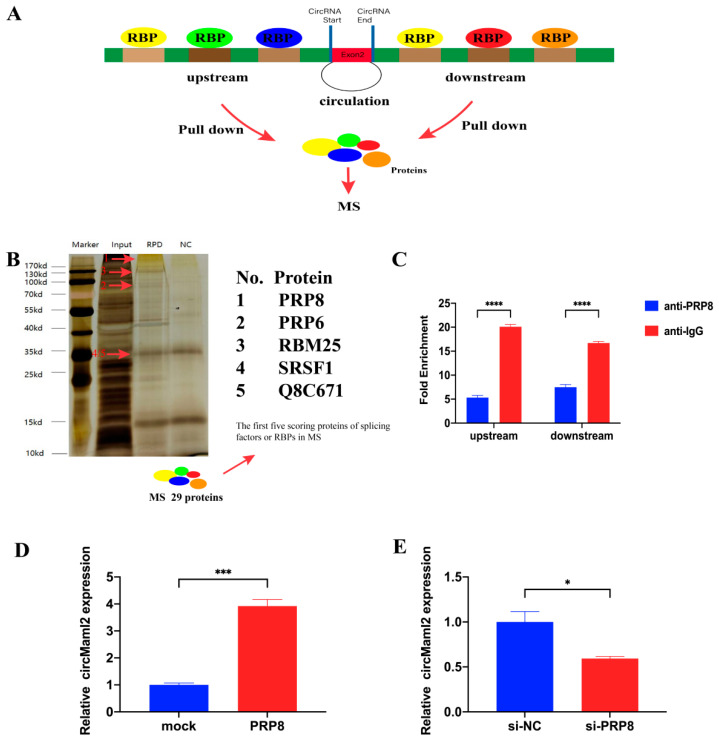
PRP8 Induces the Biogenesis of CircMaml2. (**A**) Schematic diagram of pull-down and MS and proteins in the upstream and downstream region that interact with circMaml2 pre-mRNA. (**B**) Identification of associated proteins that interact with circMaml2 pre-mRNA by silver staining and MS. (**C**) RIP assay confirmed that PRP8 could directly bind to the Maml2 pre-mRNA in MC38 cells (**** *p* < 0.0001, two-way ANOVA). (**D**,**E**) circMaml2 expression was detected in MC38 cells after PRP8 up-regulation or down-regulation by qRT-PCR (* *p* = 0.0264, *** *p* = 0.0003, *t* test). All data are presented as mean + SEM. ns, not significant. *n* = 3.

We searched relevant sequences in UCSC for the upstream and downstream regions of exon2 and then predicted the RBPs in both the upstream and downstream via catRAPID. Silver staining showed that some RBPs have the potential to combine with circMaml2 pre-mRNA. Next, pull-down and MS analyses were performed to confirm a candidate RBP from the predicted ones (Figure 7A). PRP8 was finally selected as the RBP (Figure 7B). The binding of PRP8 to Maml2 pre-mRNA was verified by RIP (Figure 7C). Moreover, the qRT-PCR analysis showed that PRP8 overexpression facilitated the expression of circMaml2 and PRP8 knockdown inhibited its expression (Figure 7D,E).

### 3.8. CircMaml2 Can Promote the Repair of Intestinal Mucosal Injury in Burned Mice 

**Figure 8:**
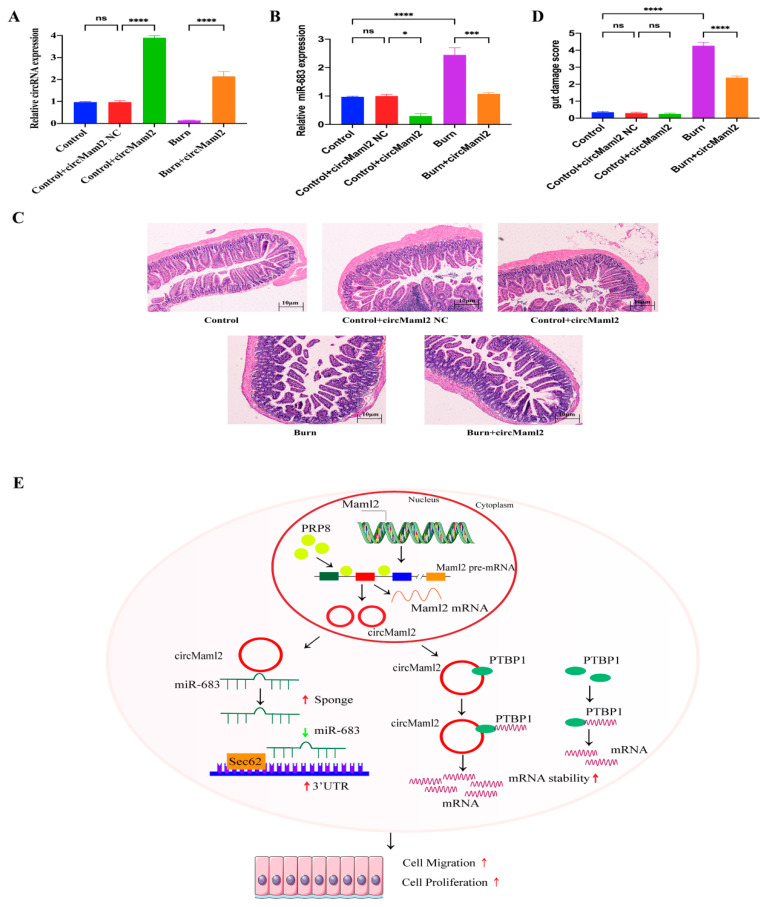
CircMaml2 Can Promote the Repair of Intestinal Mucosal Injury in Burned Mice. (**A**) Expression of circMaml2 was detected by qRT-PCR in the control group, burn group, and burn + circMaml2 group, respectively (**** *p* < 0.0001, ordinary one-way ANOVA). (**B**) The expression of miR-683 was detected by qRT-PCR in the three groups (* *p* = 0.0164, *** *p* = 0.0001, **** *p* < 0.0001, ordinary one-way ANOVA). (**C**,**D**) H&E staining and intestinal epithelium damage scoring system revealed the damage of the intestinal mucosa (**** *p* < 0.0001, ordinary one-way ANOVA, *n* = 10). (**E**) Schematic diagram of the mechanism of circMaml2 prompted by PRP8, interacting with protein PTBP1 and miR-683 to regulate cell proliferation and migration through Ebf1 and Sec62. All data are presented as mean + SEM. ns, not significant. *n* = 3.

To further confirm the effect of circMaml2 in vivo on the damaged intestinal mucosa of severely burnt mice, adenovirus overexpressing circMaml2 or circMaml2 NC was injected intraperitoneally into the gastrointestinal tract of mice before the construction of the severe burn model and the expression of circMaml2 was detected by qRT-PCR (Figure 8A). Overexpression of circMaml2 in vivo significantly inhibits the expression of miR-683 (Figure 8B). Histological examination of H&E-stained sections under light microscopy and the analysis of the intestinal epithelium damage scoring system manifested that overexpression of circMaml2 can improve the intestinal mucosal injury after severe burn (Figure 8C,D, Table 2).Therefore, these data suggest that circMaml2 might facilitate the reconstruction of the damaged intestinal mucosa via the miR-683/Sec62 and PTBP1 pathways (Figure 8E).

## 4. Discussion

Severe burns damage the skin tissue and put the whole body under critical stress. The blood in the entire body redistributes to ensure blood supply to essential organs such as the heart, brain, and kidney while supplying less blood to the intestinal tract. This aggravates intestinal mucosal ischemia and hypoxia [28]. The causes of enterogenous infection after severe burn injury mainly include the destruction of the intestinal mucosal barrier, increased intestinal permeability, and bacterial translocation [29,30]. However, the mechanism of intestinal mucosal injury and repair after severe burns is unclear. Therefore, exploring how to effectively reduce intestinal injury after severe burns and identify therapeutic targets to accelerate intestinal mucosal repair is vital. Numerous circRNAs have been demonstrated to be involved in the pathology of various diseases. However, the role of circRNAs in gastrointestinal mucosal repair requires further research.

CircRNAs are covalently closed single-stranded transcripts containing various types of RNAs [31]. CircRNAs were first produced by reverse splicing of higher eukaryotic precursor mRNA (pre-mRNA) more than 20 years ago. However, only a few circRNAs were identified then, and circRNAs were considered by-products of abnormal splicing without an underlying function [10]. RNA-seq analysis of the non-polyadenylated transcriptome revealed that circRNAs are widely expressed and have their functions [31,32,33]. Moreover, circRNA molecules can resist digestion by RNase R thanks to their special closed ring structure lacking a 5’ end cap and a 3’ ploy tail, which means they are more stable and conservative than their corresponding linear RNA molecules [32]. CircRNAs can regulate gene expression by participating in transcription and splicing, binding micro RNAs (miRNAs), interacting with proteins, and acting as templates for peptide synthesis [34]. Moreover, circRNAs can regulate cell proliferation, migration, and apoptosis and participate in the occurrence of various diseases [35,36]. CircMaml2 is a novel circRNA molecule whose characteristics and mechanistic role in diseases are unknown. According to our previous sequencing results, the expression of circMaml2 in the intestinal tract of severely burned mice was significantly decreased. This was confirmed in in vitro (cell hypoxia) and in vivo (mouse burn model) experiments in the present study.

*CircMaml2* is a 1686-nucleotide-long gene located in the autosomal chromosome 2. As circRNA molecules are formed by reverse splicing from head to tail, their structures are more stable than their linear counterparts, so even if RNase R digested them, their expression would not be influenced. In the RNase R enzyme digestion experiment, the enzyme digested the linear mRNA of the host gene Maml2, while the expression of circMaml2 remained roughly unchanged. In vitro and in vivo experiments showed that circMaml2 promoted cell proliferation, migration, and mucosal repair. CircRNAs are primarily located in the cytoplasm and perform biological functions; therefore, a nucleocytoplasmic isolation test was performed to determine the subcellular localization of circMaml2 before further analysis. The results confirmed that circMaml2 was located in the cytoplasm.

CircRNAs interact with RBPs to exert their biological functions [37,38]. RBPs play a vital role in the post-transcriptional regulation of RNA and are involved in tissue development and disease [39]. RBPs contain an RNP complex, which binds RNA sequences by interacting with specific cis-regulatory elements. They participate in the occurrence, translation, transcription, and transportation of target genes and affect the expression and function of target RNAs. More than 800 RBPs have been found in the human genome, containing almost 40 domain motifs [23]. RBPs are widely expressed in different tissues, but some exhibit tissue-specific expression or are produced under pathological conditions; these can be explored as biomarkers of diseases [40,41].

In our study, we first conducted a pull-down experiment and analyzed the pull-down protein products by MS to identify RBPs binding to circMaml2. The two experiments revealed 301 proteins that could bind to circMaml2. According to the mass spectrum score and the function of pull-down proteins, we selected PTBP1 as the candidate RBP that received the highest score of the proteins related to cell proliferation and migration. The binding of PTBP1 and circMaml2 was verified via an RIP test. After binding, circRNAs interact with RBPs and affect each other functionally. In this study, overexpression of circMaml2 did not affect the expression of PTBP1 at the molecular level, as seen by both qRT-PCR and Western blot analyses.

PTBP1 is expressed in almost all types of cells and belongs to the PTB family, including PTBP2 and PTBP3, as well as the subfamily of heteronuclear ribonucleoproteins (hnRNPs) as an RBP. Its gene is located on human chromosome 19p13.3 [41,42,43,44]. Structurally, PTBP1 is 531 amino acids long with an N-terminal nuclear shuttle domain and four repetitive RNA recognition motifs used to bind RNA and participate in the RNA-binding process [45]. As an RBP, the function of PTBP1 is to bind to RNA and interact with different RNAs (such as lncRNA and circRNA). Moreover, PTBP1 participates in the post-transcriptional regulation of mRNA, including splicing, translation, stabilization, and localization. The current study has not explored the binding of PTBP1 and circMaml2 and how the complex regulates intestinal mucosal injury and repair after-burn.

To explore the mRNAs regulated by binding of PTBP1 to circMaml2 in the intestinal mucosa after severe burn and its possible mechanisms, we predicted the mRNAs regulated downstream of PTBP1 through the website http://starbase.sysu.edu.cn (accessed on 29 August 2022). Several mRNAs related to the function of circMaml2 and PTBP1 were selected by sorting the number of binding regions, and qRT-PCR demonstrated that the increase in Ebf1 expression was the most stable.

Ebf1 is an important regulator of transcription that determines the development of early B lymphocytes. It significantly promotes cell proliferation, cell differentiation, the survival rate of pre-B cells, and the formation of B lymphocytes through the mediation of many inflammatory and metabolic pathways such as PI3K/Akt and mitogen-activated protein kinase.

The RIP experiment was initially performed to confirm the binding between PTBP1 and Ebf1 and explore the effect of PTBP1 on Ebf1. The actinomycin D assay showed that PTBP1 could regulate the post-transcriptional modification of Ebf1 mRNA and improve its stability. Western blot analysis verified the positive effect of PTBP1 on Ebf1 at the protein level. Similar methods were applied in MC38 cells co-transfected with circMaml2 and PTBP1 and showed that circMaml2 and PTBP1 in promoting Ebf1 was more prominent than that of PTBP1 alone. Thus, we can assume that circMaml2 promotes the function of PTBP1. In the cell function test, circMaml2 enhanced the stability of Ebf1 by promoting PTBP1 expression, thereby increasing the proliferation and migration of cells. As for the further interaction between circRNAs and RBPs, it concludes numerous complex mechanisms. For instance, circRNA can adsorb RBP as a sponge to reduce the expression of free RBP as well as the physiological effect of RBP [38], or it can bind with RBP to protect it from protease degradation (such as ubiquitin), thereby increasing its stability. This, in turn, maintains the protein expression level of RBP and promotes its effect [37]. CircRNAs also act as scaffolders that physically combine RBPs with downstream targets (mRNA or protein) to enhance the function of RBPs [39,40,41]. In this study, we mainly investigate increasing the stability of RBP to improve the biological effect of RBP.

CircRNAs also act as a microRNA sponge. As an imperative non-coding small-molecule RNA, miRNA often participates in post-transcriptional regulation and inhibits the expression of its target gene by inducing the binding between RNA-induced silencing complex and the site complementary to the miRNA seed sequence in the 3’ UTR of mRNA, which may lead to the degradation of mRNA or inhibit mRNA translation [46]. The TargetScan and miRanda prediction software analysis revealed that the miRNA sequence was complementary to circMaml2. qRT-PCR showed that the expression of miR-93-3p and miR-683 was increased and was verified by a double luciferase reporter gene assay, in which circMaml2 was found to be a molecular sponge of miR-93-3p and miR-683. As miR-93-3p has already been investigated in detail, we chose miR-683 for further research. The relevant characteristics and biological functions of miR-683 have not been reported.

Sec62 in mammals can form a complex with Sec61, assisting protein translocation in the endoplasmic reticulum membrane. It also interacts with Sec63, an endoplasmic reticulum protein containing the lumen J domain [47,48]. Given the high positive rate of Sec62, it is regarded as a possible target gene for prostate, lung, and thyroid cancer. A study showed that, by silencing Sec62, the migration and invasion of prostate cancer cells were significantly reduced with minimal effect on cell viability.

In our research, Sec62 was predicted as a possible target of miR-683 through the biological information analysis website, and the combination of the two was verified through the double fluorescein reporter gene experiment. qRT-PCR and Western blot analyses showed that miR-683 reduced Sec62 expression, while the addition of circMaml2 reversed the negative effect of miR-683. Similarly, it was verified in cell function tests that circMaml2 may regulate miR-683 and its downstream Sec62 through the “sponge adsorption” mechanism to affect cell proliferation and migration.

After discussing the mechanism of circMaml2, we studied the biogenesis of circMaml2. CircRNAs are derived from pre-mRNAs. The biogenesis and emerging roles of circular RNAs and RBPs have been reported to regulate circRNA function and circRNA biogenesis [21,38,49,50,51]. One classical example affecting biogenesis is QKI, which upregulates many circRNAs during epithelial to mesenchymal transition (EMT) in humans via pre-mRNA binding. As QKI forms dimers, it was proposed that QKI may bind to two flanking introns and bring the circularized exons closer together, resulting in the increased production of circRNAs [51,52].

Therefore, in our study, we speculated that some RBPs could regulate the biogenesis of circMaml2. Thus, we predicted some possible RBPs using catRAPID. Pull-down and MS analyses confirmed PRP8 as a significant RBP, while RIP testified the result in the reverse direction. Furthermore, qRT-PCR showed that the amount of PRP8 affected the expression of circMaml2.

In conclusion, this study shows that circMaml2 enhances the biological function of PTBP1 by binding to it and improves the mRNA stability of Ebf1. In addition, circMaml2 can act as a “molecular sponge”, acting as a ceRNA in MC38 cells and regulating the effect of miR-683 on the target gene Sec62 by combining it with miR-683. PRP8 regulates the biogenesis of circMaml2. Furthermore, circMaml2 was also expressed in vivo and manifested its mechanism via PTBP1 and miR-683, which could relieve the injury of burnt intestinal mucosa of mice. Through these pathways, circMaml2 plays a significant role in cell proliferation and migration and further repair and reconstruction of the damaged intestinal mucosa. In addition, in our research, we have simplified the migration assays of would healing, in which we took out the effect of some extracellular factors like type IV collagen and laminin. Thus, it may be the limitation of our experiments and we will try to address that in our future study. Therefore, we have preliminarily described the relationship among circMaml2, PTBP1, and miR-683, providing an experimental basis for possible gene therapy for intestinal mucosal injury and repair after clinical burns.

## 5. Conclusions

CircMaml2 can enhance PTBP1 function by binding to it and regulating downstream mRNA Ebf1 to promote intestinal mucosal repair, while binding miR-683 to target sec62 promotes intestinal mucosal repair. Moreover, circMaml2 can be promoted by PRPB. The results may contribute to illuminating the mechanism of promoting intestinal mucosal repair and provide a basis for clinical circRNA gene therapy and small molecule chemotherapy.

## Figures and Tables

**Table 1:** Relative primers.

Genes	Primers (5′-3′)
CircMaml2	Forward: GGGTCAGCAGAGCCAGAGGAG; Reverse: GAGAGCCTGAAGTGCCTTGTGTC
Maml2	Forward: GAGCCTGGTAATGACGACTGGATG; Reverse: GAGCCTGCGGATCATCTTCCTTC
PTBP1	Forward: AGCCAGCCTTCACTAGACCAGAC; Reverse: CAGCAGCAGCAGCAGCAGAC
Ebf1	Forward: AGAAGACAGCAGGTCCACATCCTC; Reverse: ACTTGTAATGTGCGGCTGCCTTC
Gpc6	Forward: GGAGTCACCCAAAGTCCCAC; Reverse: AGAGATACAGGGCCGAGTCC
Lpp	Forward: TGGATGCTTGCGGTCTCATTCAC; Reverse: CAGAGCCACAATGCGGACAGTC
Auts2	Forward: CGAAGACGACCCGAAAGCAGAC; Reverse: AGGCACGGCAGATGTAGGAGAG
Smyd3	Forward: GATGCCAACATACGAGCCTCCTAAG; Reverse: TACAGAAGGTCCACACAGCAAACAC
Dock1	Forward: CGAAGACGACCCGAAAGCAGAC; Reverse: AGGCACGGCAGATGTAGGAGAG
St6galnac3	Forward: TTTATTCTGCTCTTCGTTGT; Reverse: TGCTCTGAGGATTCTCTGGT
miR-683	Forward: TGCCCACTCTACCCATTGATTGC
Sec62	Forward: GATGAGGAGGATGACGACAAAGATGG; Reverse: ATGACCGCCTTTCTCTGGATTGAAC
Klhdc8a	Forward: GGGCAGTTGTGTAGGAAGGAAGC; Reverse: AGGTCAGGAGAAGGTGGCAGAAG
Smurf1	Forward: CGATGAGGAGAGGAGAGCCAGAC; Reverse: GCAGAGCCTTGAAGCCTTGGAG
PRP8	Forward: CGAGTCTGGCTGTTCTTTATGC; Reverse: ATGTACGGACCGTCCTTTAAGTAG
GAPDH	Forward: TTCAACGGCACAGTCAAG; Reverse: CACCCCATTTGATGTTAGTG
U6	Forward: CCTGCTTCGGCAGCACA

**Table 2:** Intestinal epithelium damage scoring system.

Score	Histologic Characteristic(s)
1	Only lost the tip of the villus.
2	50% villus loss
3	The entire villus disappeared, but the crypts were preserved.
4	Completely lost the epithelium.

## Data Availability

The datasets used and analyzed during the current study are available from the corresponding author upon reasonable request.
